# Grade I and Grade II Penile Strangulation From a Plastic Bottleneck

**DOI:** 10.7759/cureus.71306

**Published:** 2024-10-12

**Authors:** Robin Z Cheng, Wei-Shin Lu, Srigita K Madiraju, Bradley Buck, Ahmed El-Zawahry

**Affiliations:** 1 Urology, The University of Toledo College of Medicine and Life Sciences, Toledo, USA; 2 Urology, Robotic and Minimally Invasive Urologic Surgery Institute of Northwest Ohio, Toledo, USA

**Keywords:** corporal aspiration, penile strangulation, penile strangulation grading, ring cutter, rotating saw

## Abstract

In this case report, we present two clinical cases of penile strangulation caused by a bottleneck at the base of the penis. In the first case, corporal aspiration was used to decrease the blood that is trapped in the corpora due to the constriction. A rotating saw was used to incise the bottleneck in a full-thickness manner. The plastic bottleneck was successfully incised and extricated with the rotating saw while the penile shaft was protected. This technique is novel and unique, as there are no prior instances in the literature where both corporal aspiration and a rotating saw were used simultaneously. In the second case, penile strangulation was resolved with a ring cutter, which allowed the bottleneck to be extricated without causing further laceration. Both the combined use of corporal aspiration and a rotating saw, as well as the utilization of a ring cutter, were efficacious approaches that led to immediate improvement in the patients.

## Introduction

Penile strangulation is a urological emergency that requires urgent intervention to remove the constricting device to prevent vascular ischemia and eventual necrosis if untreated [1]. Often, patients place various nonmetallic and metallic objects on the penis for autoerotic purposes or to increase sexual performance [1,2]. Entrapment of the penis by a constricting object can cause obstruction of arterial supply and venous return in the corpora cavernosum, resulting in compartment syndrome of the penis. End-stage progression of the strangulation can cause gangrene, necrosis, and amputation of the penis. Patients with penile incarceration lasting more than 72 hours are more likely to experience higher-grade injuries compared to those who are treated more promptly [3]. Therefore, it is necessary to expeditiously relieve the strangulation.

Many cases of penile strangulation are reported every year and frequently described as isolated case reports or small series. In this case report, we present two cases of penile entrapment due to bottlenecks that differ in surgical approach. We categorize these two cases of penile strangulation in a grading system as well as illustrate a novel interventional method of treatment.

## Case presentation

Case 1

**Figure 1:**
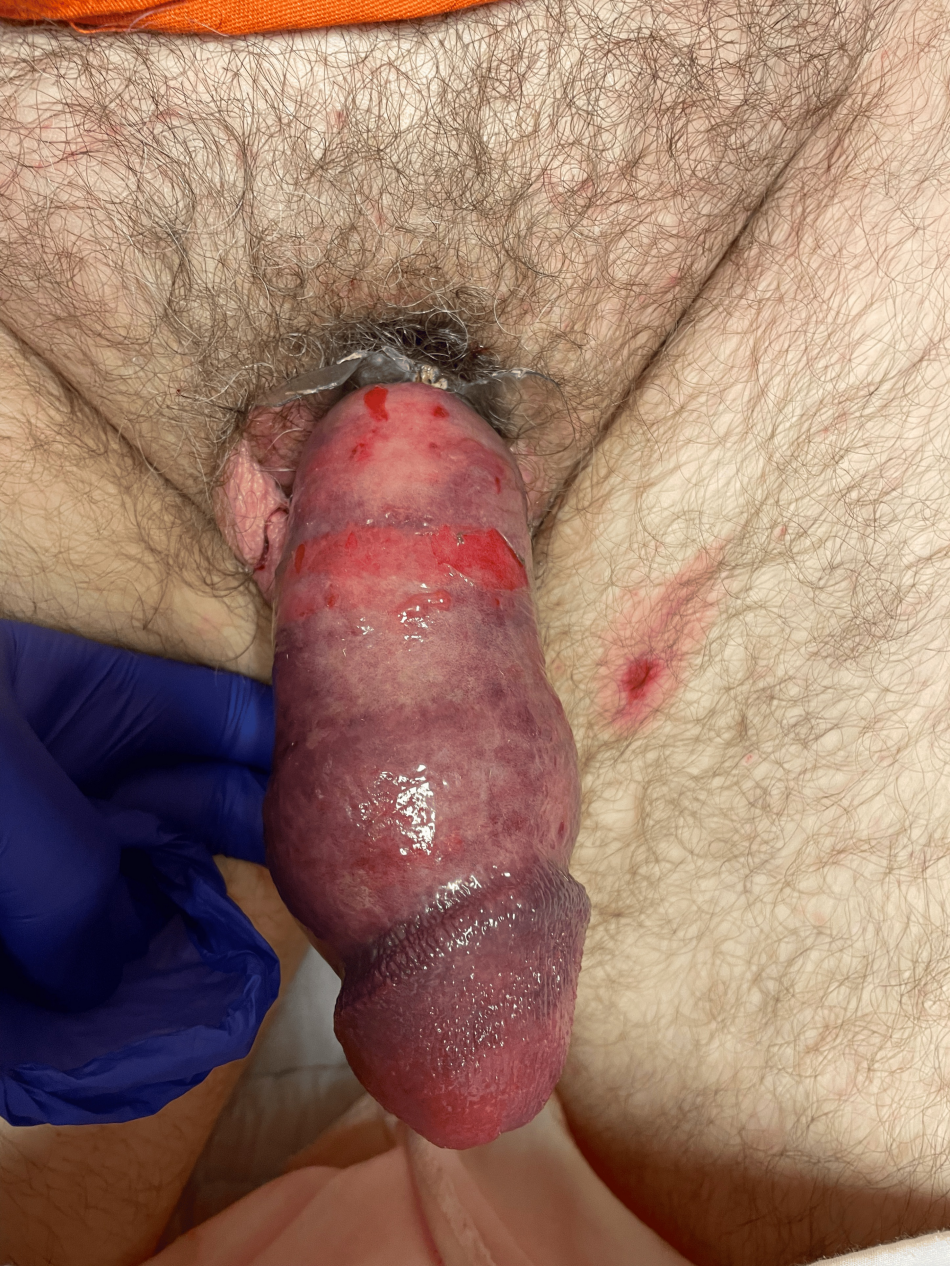
Preoperative photograph demonstrating the mouth of a plastic bottle constricting at the base of the shaft of the penis, causing extensive edema.

**Figure 2:**
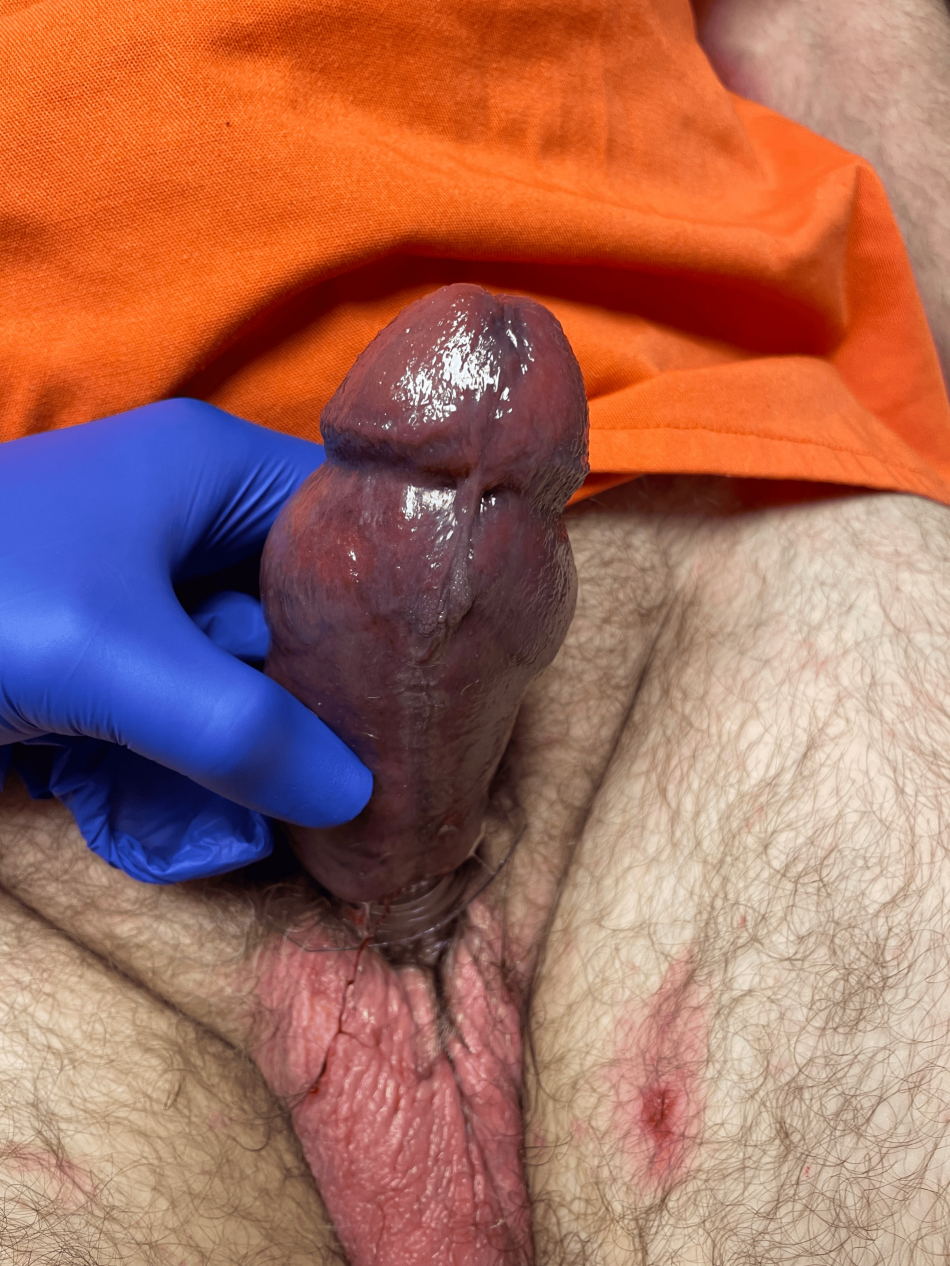
Preoperative photograph demonstrating severe ecchymosis, ischemia, and edema present on the penis.

A 58-year-old Caucasian male presented with a two-day history of a foreign body constricting around the base of his penile shaft. The patient had placed a lotion bottle around the penis until the opening of the plastic bottle constricted around the base of his penis. The patient developed edema around the base, causing more constriction. Outside hospital staff failed to remove the bottleneck after successfully removing the rest of the bottle. The patient was then transferred to our tertiary center with extensive edema (Grade II) [4]. On examination, he had a swollen and bruised penis with the mouth of a lotion bottle constricted around the base of the shaft of the penis (Figure 1). The patient endorsed loss of sensation on the penis distal to the site of constriction. Severe edema, ecchymosis, numerous lacerations, and ischemia of the penis were observed (Figure 2).

Under general anesthesia, two 16-gauge needles were inserted into the patient's corpus cavernosum through the glans to drain the corpora. Penile swelling was reduced with drainage of ischemic blood and intermittent compression of the penile shaft until the swelling diminished, decreasing the shaft diameter.

Two pieces of umbilical tape, with the assistance of a hemostat, were used to tie around the base of the penis to maintain compression and create space between the bottleneck and penile shaft. A hand-held rotating saw was used to cut along the bottleneck longitudinally while the penile tissue was protected until the cut was complete. The bottleneck was pulled apart and extricated from the penile shaft. A 2-cm laceration extending to the subcutaneous tissue was seen on the right ventral side of the penile shaft, and the skin was closed using a 3-0 chromic suture. A 22 French flexible cystoscope with a 30-degree lens was then inserted in the patient's urethra and advanced into the bladder, and no injuries in the urethra or bladder were seen. However, the bladder appeared to have a large capacity and high residual volume, indicating the patient had retention due to the constriction. A 16 Fr Foley catheter was inserted per the urethra, which drained 700 cc of clear yellow urine, indicating that the patient was in urinary retention. The penis was cleaned, and bacitracin ointment was applied. Bandage roll dressing (Kerlix™, Covidien, Dublin, Ireland) was applied circumferentially, and self-adherent wrap with latex (Coban™, 3M, St. Paul, MN) was placed around the bandage roll dressing.

**Figure 3:**
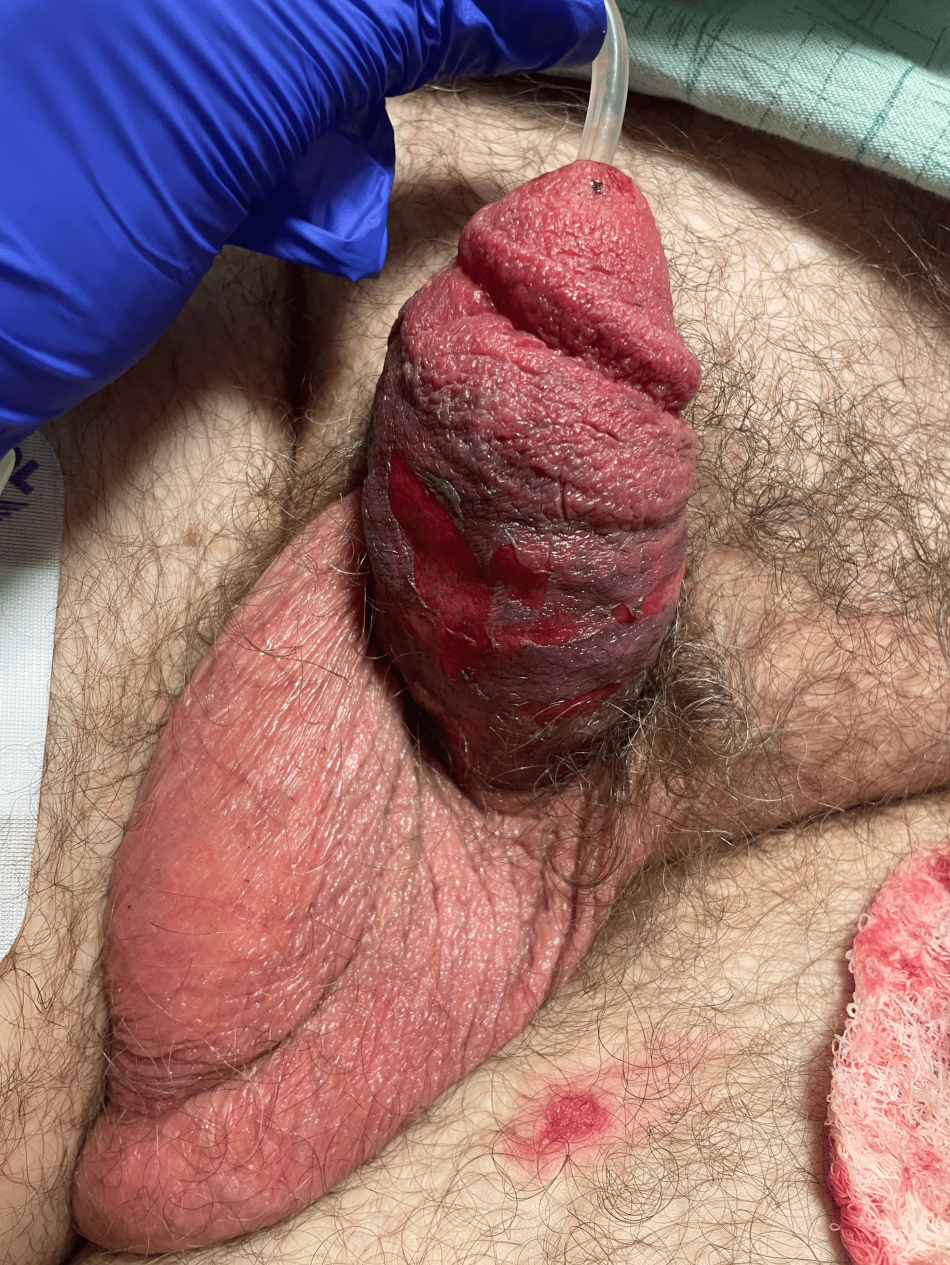
Postoperative photograph of the ventral side of the penis demonstrating a significant decrease in ecchymosis and edema of the penis.

**Figure 4:**
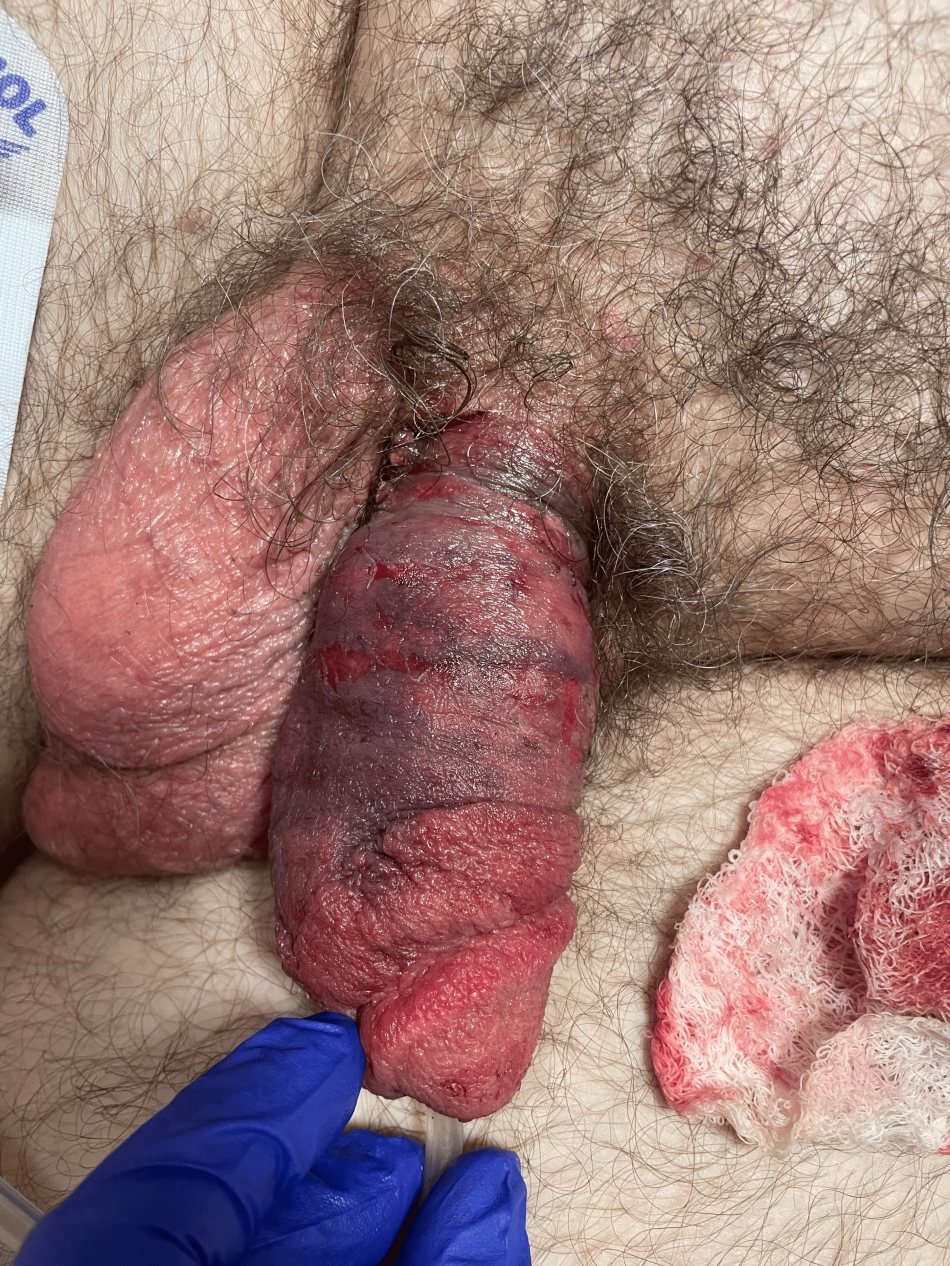
Postoperative photograph of the dorsal side of the penis demonstrating a substantial decrease in ecchymosis and swelling of the penis.

The dressing was removed approximately 12 hours later, and the edema and ecchymosis improved (Figures 3, 4). The patient was started on oral tamsulosin 0.4 mg daily for an eventual void trial. On postoperative day one, the patient was afebrile and hemodynamically stable. The patient's Foley catheter was removed, and he successfully passed a void trial. The patient was discharged in stable condition. The patient was lost to follow-up.

Case 2

**Figure 5:**
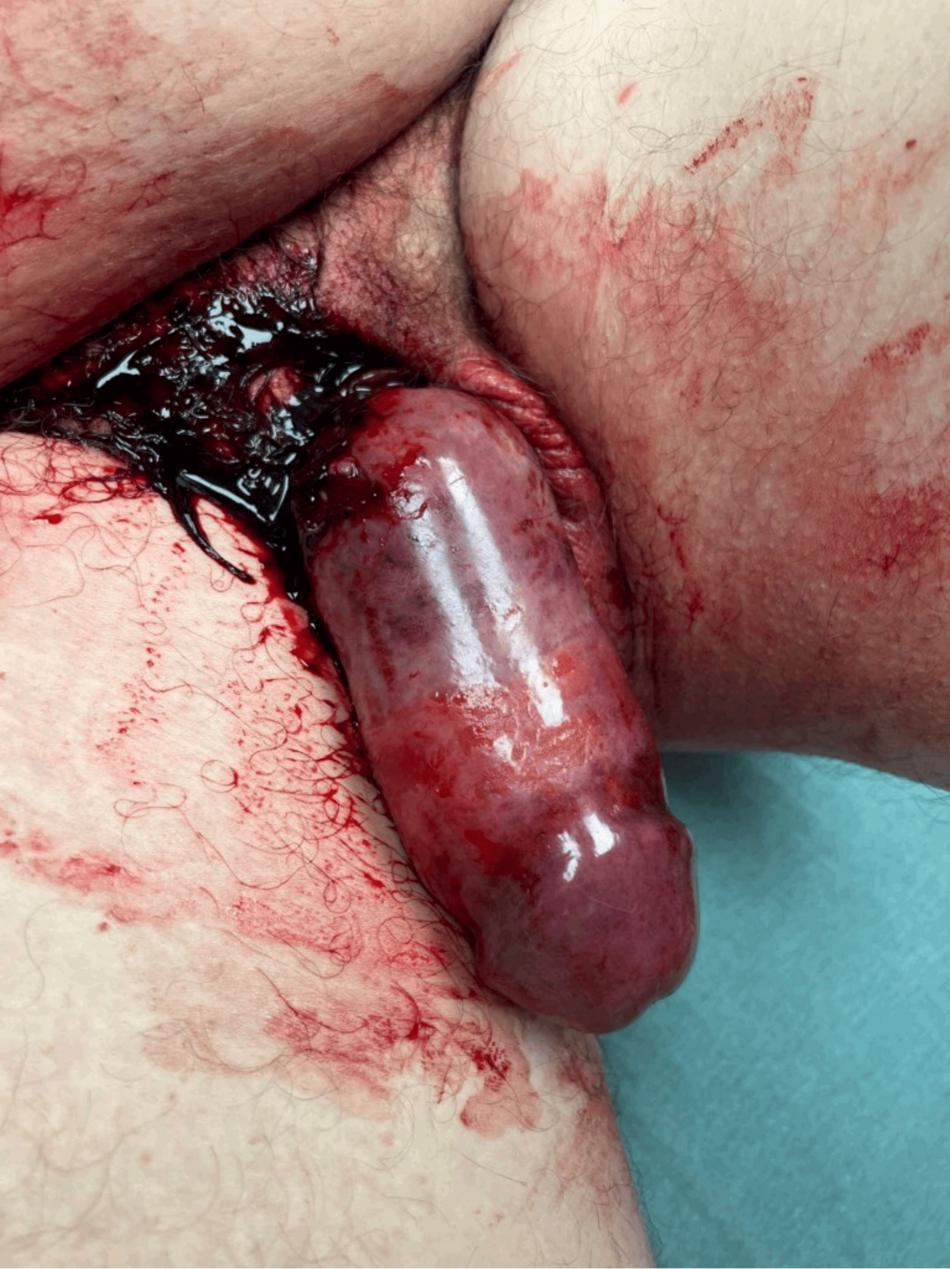
Preoperative photograph demonstrating a plastic bottleneck constricting the base of the penis, accompanied by penile swelling and superficial laceration.

A 47-year-old male presented with penile pain, swelling, and strangulation secondary to inserting his penis into a plastic bottle and being unable to remove the bottle for three hours. The patient had attempted to cut the bottle off but was unable to remove the bottleneck (Figure 5). This was stuck at the base of the penis and led to penile swelling and superficial laceration distal to the area of strangulation (Grade I) [4].

The patient was given 0.3 mg/kg ketamine for 15 minutes. A ring cutter was used to incise the bottleneck multiple times until it was cut in its entirety. The bottleneck was pried open and gently removed from around the penile shaft without accidentally closing the bottleneck midshaft or causing more lacerations. Two dorsal penile superficial lacerations were noted. 20 mL of 1% lidocaine injection was administered for a penile ring block. The lacerations were then sutured with 4-0 chromic. The penile swelling dramatically reduced with no gross areas of necrosis. A light pressure dressing was placed with self-adherent wrap with latex (Coban™, 3M) that was removed after several hours. The patient was able to void with a low post-void residual. The patient followed up with his primary care physician three weeks after the procedure. On physical exam, the penis showed no erythema, tenderness, or swelling; the penile laceration repairs appeared clean, dry, and healing well.

## Discussion

Penile strangulation cases often differ in severity. Our two cases are prime examples, with one case presenting penile swelling after a three-hour entrapment and another case presenting bruising and swelling after a two-day history of penile strangulation. Multiple grading systems have been proposed to classify penile strangulation (Table 1). Sawant et al. proposed a grading system that grades penile strangulation by the division of the corpus spongiosum and constriction of the corpus cavernosum [5]. Bhat et al. proposed a grading system that classifies based on the sensation of the penis [6]. We prefer to use a simple classification system proposed by Dawood et al. that depends on a quick physical examination to help all the staff understand the type of injury [4]. This grading system seeks to be simpler and more accurate, as both previous systems are either not easily identifiable by examination or require accurate patient-reported symptoms.

**Table 1:** A summary of penile strangulation grading systems.

Study	Grade 0	Grade I	Grade II	Grade III	Grade IV	Grade V
Sawant et al. [5]	No urethral injury	Partial division of corpus spongiosum with urethrocutaneous fistula	Complete division of corpus spongiosum with constriction of corpus cavernosum	Gangrene and amputation	-	-
Bhat et al. [6]	-	Edema distal to the penis	Grade I and decreased distal penile sensation	Grade I and loss of distal penile sensation	Grade III and complete separation of corpus cavernosa	Gangrene or amputation
Dawood et al. [4]	-	Superficial injury with distal edema	Injury to the corpora or urethra	Gangrene, amputation, fistula, or separation of corpora	-	-

In our first case, the patient presented with a two-day history of a bottleneck constricted around the base of his penis, causing penile strangulation resulting in penile swelling and ecchymosis due to the corpora cavernosa being engorged with trapped blood, penile ischemia as evidenced by the dark aspirated blood, and loss of penile sensation indicating neurological compromise. Using both our system as well as Bhat's, this case would be considered a Grade II penile strangulation. It was successfully resolved with the extrication of a plastic bottleneck that was constricted around the base of the penis, using intracorporal aspiration and a rotating saw.

Our second case presented similarly with a three-hour history of penile swelling and pain. As this case mostly presented with superficial injury and distal edema, our second case would be considered a Grade I penile strangulation. Due to a shorter history of symptoms and less severe presentation, this case was resolved easily through extrication of the bottleneck with a ring cutter.

In literature, a case series demonstrated patients who have placed metal bearing around the base of the penis that was removed using ligature silk string along with corporal glandular aspiration [7]. A case series featured several instances of penile strangulation, including a wedding ring around the penis treated with a metal ring cutter, a steel cuff cut with a steel saw, a bullring cut with a bolt cutter, a hammerhead cut with an electrical steel saw, and a plastic bottleneck cut by a scalpel [8]. There has been a report of penile strangulation caused by a metal ring resolved by utilizing a ring cutter, a cable wire cutter, and a bone cutter [9-11].

There have been prior instances in literature where electrical saws and high-speed drills were used to relieve penile strangulation caused by the constriction of a foreign object [8,12,13]. Additionally, there have been cases in which corporal aspiration was performed to decrease corporal swelling and facilitate the removal of the constricting device [7]. However, there have been no documented instances in literature in which both a rotating saw and corporal aspiration were used simultaneously in a case, as was seen in our first case. Through the judicious use of corporal aspiration to decrease the intracorporal volume and pressure, a hemostat and umbilical tape to create space and protect the penile shaft, and the rotating saw to make a full-thickness incision through the bottleneck, the constricting device was safely and successfully extricated. Our second case endorses the use of a ring cutter, which has been reported to be effective in treating penile strangulation in literature [8,9].

## Conclusions

Penile strangulation is a urological emergency that requires immediate assessment and treatment. There are various approaches to treating cases of penile strangulation, and interventional methods are employed on a case-by-case basis. We demonstrated one case of penile strangulation that was surgically resolved with intracorporal aspiration, followed by mechanical cutting of the constricting device with a rotating saw, and a second case that was relieved with a ring cutter. It is helpful to categorize penile strangulation in a grading system to ease communication regarding the severity of the case. This could allow for expedited treatment and better care coordination for patients.

## References

[1] Ivanovski O, Stankov O, Kuzmanoski M (2007). Penile strangulation: two case reports and review of the literature. J Sex Med.

[2] Sarkar D, Gupta S, Maiti K, Jain P, Pal DK (2019). Penile strangulation by different objects and its removal by the modified string method: management of four cases with review of literature. Urol Ann.

[3] Silberstein J, Grabowski J, Lakin C, Goldstein I (2008). Penile constriction devices: case report, review of the literature, and recommendations for extrication. J Sex Med.

[4] Dawood O, Tabibi S, Fiuk J, Patel N, El-Zawahry A (2020). Penile ring entrapment - a true urologic emergency: grading, approach, and management. Urol Ann.

[5] Sawant AS, Patil SR, Kumar V, Kasat GV (2016). Penile constriction injury: an experience of four cases. Urol Ann.

[6] Bhat AL, Kumar A, Mathur SC, Gangwal KC (1991). Penile strangulation. Br J Urol.

[7] Noh J, Kang TW, Heo T, Kwon DD, Park K, Ryu SB (2004). Penile strangulation treated with the modified string method. Urology.

[8] Perabo FGE, Steiner G, Albers P, Muller SC (2002). Treatment of penile strangulation caused by constricting devices. Urology.

[9] Low LS, Holmes M (2018). The GEM ring cutter: an effective, simple treatment of penile strangulation caused by metal rings. Urol Case Rep.

[10] Neupane D, Singh SK, Kafle A, Chaudhary S, Subedi SS, Chhetri S (2021). Penile strangulation with a plastic bottle neck: intervened by an atypical instrument: a case report. Int J Surg Case Rep.

[11] Rohith G, Dutta S, G S S (2020). A rare case of penile strangulation by a hard plastic bottleneck. Cureus.

[12] Bahadori A, Bray G, Khan M (2023). Penile strangulation secondary to a plastic bottle neck. Urol Case Rep.

[13] Huang JK, Holt D, Philp T (1997). Penile constriction by foreign bodies: the use of a dental drill. Br J Urol.

